# Working cancer survivors’ physical and mental characteristics compared to cancer-free workers in Japan: a nationwide general population-based study

**DOI:** 10.1007/s11764-020-00984-7

**Published:** 2021-01-12

**Authors:** Atsuhiko Ota, Yuanying Li, Hiroshi Yatsuya, Kozo Tanno, Kiyomi Sakata, Kazumasa Yamagishi, Hiroyasu Iso, Nobufumi Yasuda, Isao Saito, Tadahiro Kato, Kazuhiko Arima, Yoko Sou, Taichi Shimazu, Taiki Yamaji, Atsushi Goto, Manami Inoue, Motoki Iwasaki, Norie Sawada, Shoichiro Tsugane, for the JPHC-NEXT Study Group

**Affiliations:** 1 Department of Public Health, Fujita Health University School of Medicine, 1-98 Dengakugakubo, Kutsukake-cho, Toyoake, Aichi 470-1192 Japan; 2 Department of Public Health and Health Systems, Nagoya University Graduate School of Medicine, Nagoya, Japan; 3 Department of Hygiene and Preventive Medicine, School of Medicine, Iwate Medical University, Iwate, Japan; 4 Department of Public Health Medicine, Faculty of Medicine, and Health Services Research and Development Centre, University of Tsukuba, Tsukuba, Japan; 5 Ibaraki Western Medical Center, Chikusei, Japan; 6 Department of Social and Environmental Medicine, Osaka University Graduate School of Medicine, Osaka, Japan; 7 Department of Public Health, Kochi University Medical School, Kochi, Japan; 8 Department of Public Health and Epidemiology, Faculty of Medicine, Oita University, Oita, Japan; 9 Center for Education and Educational Research, Faculty of Education, Ehime University, Matsuyama, Japan; 10 Department of Public Health, Nagasaki University Graduate School of Biomedical Sciences, Nagasaki, Japan; 11 Ken’ou Healthcare Office, Nagasaki Prefectural Government, Nagasaki, Japan; 12 Epidemiology and Prevention Group, Center for Public Health Sciences, National Cancer Center, Tokyo, Japan; 13 Department of Health Data Science, Yokohama City University Graduate School of Data Science, Yokohama, Japan

**Keywords:** Work, Cancer survivors, Self-rated health status, Physical functional capacity, Depressive symptoms, Happiness

## Abstract

**Purpose:**

This study compared working cancer survivors’ self-rated health status (SRHS), physical functional capacity, depressive symptoms, and happiness to those of cancer-free workers.

**Methods:**

A nationwide general population-based cross-sectional study on a sample of Japanese was conducted. Prevalence of deteriorated SRHS, restricted physical functional capacity, depressive symptoms, and perceived happiness were compared between working cancer survivors and cancer-free workers with multivariable logistic regression analysis adjusted for age and sociodemographic and health-related backgrounds.

**Results:**

Of the 28,311 male and 26,068 female workers, 977 (3.5%) and 1267 (4.9%) were cancer survivors, respectively. Working cancer survivors reported deteriorated SRHS more frequently than cancer-free workers: 21.3% vs. 13.8%, multivariable-adjusted odds ratio (95% confidence interval), 1.64 (1.39–1.95) for men, 23.8% vs. 17.5%, 1.34 (1.16–1.54) for women. Restricted physical functional capacity was reported more frequently in working cancer survivors than cancer-free workers: 6.8% vs. 2.6%, 1.76 (1.34–2.32) for men, 4.9% vs. 2.0%, 2.06 (1.56–2.71) for women. No significant difference was found for depressive symptoms: 21.6% vs. 22.9% in men, 30.0% vs. 28.5% in women. Working cancer survivors felt happiness more frequently than cancer-free survivors in men (77.3% vs. 71.7%, 1.21 (1.01–1.45)) but not in women (76.1% vs. 74.9%).

**Conclusions:**

Working cancer survivors had worse SRHS and more restricted physical functional capacity than cancer-free workers. In men, working cancer survivors felt happiness more frequently than cancer-free workers.

**Implications for Cancer Survivors:**

Continuous support to improve cancer survivors’ SRHS and physical functional capacity would be necessary even while they are working.

**Supplementary Information:**

The online version contains supplementary material available at 10.1007/s11764-020-00984-7.

## Introduction

In Japan, the age-adjusted cancer mortality rate has been decreasing over the past few decades; the latest 5-year relative survival rate for cancer is estimated to be 62.1% [1]. Consequently, questions on how to improve health and well-being of cancer survivors have attracted more clinical attention. Researchers addressed cancer survivors’ health-related quality of life (HRQOL). Existing research generally revealed the prognostic ability of deteriorated HRQOL in cancer survivors [2, 3]. Individual-level HRQOL includes physical and mental health perceptions and their correlates, such as health risks and conditions and functional status [4]. Self-rated health status (SRHS), physical functional capacity, depressive symptoms, and happiness (regarded as affective well-being [5, 6]) form a component of HRQOL. Cancer survivors claimed deteriorated SRHS [6, 7], restricted physical functional capacity [6, 7], depressive symptoms [6, 8], and unhappiness [6] more frequently than the cancer-free individuals. Deteriorated SRHS [9, 10], decreased physical activity [11, 12], and depression [13] worsened the prognosis of cancer survivors.

There is limited evidence regarding working cancer survivors’ SRHS, physical functional capacity, depressive symptoms, and happiness. Working is a form of societal involvement for the working-age; thus, the Japanese government and employers must assist working cancer survivors in finding a balance between work and treatment under the Cancer Control Act [1]. The return-to-work rates among cancer survivors reportedly ranged from 53.8 to 95.2% in Japan [14]. Compared to cancer survivors who were not working, those who were working were more likely to have better SRHS [15, 16] and physical functional capacity [17, 18], and a lower prevalence of depression [19–21].

It was not well addressed whether the prevalence of poor SRHS, restricted physical functional capacity, depressive symptoms, and happiness differed between working cancer survivors and cancer-free workers. In a Japanese study, compared to cancer-free workers, working cancer survivors showed a significantly higher prevalence of deteriorated SRHS in men, of restricted physical functional capacity in both men and women, but not of unhappiness in either men or women [22]. Similarly, working cancer survivors’ SRHS was worse than that of cancer-free workers in Norway [23]. It is controversial whether the prevalence of depressive symptoms differed between working cancer survivors and cancer-free workers [24]. Few studies examined the differences in happiness between working cancer survivors and cancer-free workers.

Therefore, we conducted a cross-sectional analysis to compare the prevalence of deteriorated SRHS, restricted physical functional capacity, depressive symptoms, and happiness between working cancer survivors and cancer-free workers at the national level, using a nationwide general population sample.

## Methods

### Study design and sample

We conducted a cross-sectional investigation using baseline data from the Japan Public Health Center-based Prospective Study for the Next Generation (JPHC-NEXT Study). Details of its design and participants are reported elsewhere [25]. Eligible participants were 261,939 residents (130,602 men and 131,337 women) aged 40–74 in 16 municipalities of seven prefectures across Japan. The baseline survey was conducted between 2011 and 2016. Of the 114,105 individuals (52,566 men and 61,539 women) who responded to the questionnaire, 64,960 individuals (33,632 men and 31,328 women) fulfilled the inclusion criteria of the present study: aged 40–65 years and workers at baseline. Workers were defined as those who did not declare themselves to be an unemployed individual or a homemaker. After excluding 10,581 individuals with missing responses for any of the study variables, we analyzed data from 54,379 individuals (28,311 men and 26,068 women).

### Study variables

All data were collected through a questionnaire. Participants were asked about their cancer history, SRHS, physical functional capacity, depressive symptoms, happiness, and sociodemographic and health-related backgrounds.

### Definition of working cancer survivors and cancer-free workers

We used two questions to define the working cancer survivors and cancer-free workers. The first question referred to working status. The participants chose their occupation from the following options: unemployed, homemaker, professional or technical work, administrative work, clerk, sales, service, security, agriculture or fishery, transportation or telecommunications, industrial operation/management, other. Those who did not choose unemployed or homemaker were regarded as a worker. Those who chose the options of professional or technical work, administrative work, clerk, sales, or service were regarded as white-collar workers, while those who chose security, agriculture or fishery, transportation or telecommunications, industrial operation/management, or other were regarded as blue-collar workers. The second question asked whether they had been diagnosed with and treated for cancer. The applicable options included “stomach cancer,” “colorectal cancer,” “lung cancer,” “liver cancer,” “breast cancer,” “prostate cancer,” and “other cancer.”

Participants who reported to be a worker and had been diagnosed with and treated for cancer were regarded as working cancer survivors. Participants who reported to be a worker and had not been diagnosed with cancer were regarded as cancer-free workers.

### SRHS

Participants responded to the question, “What do you think of your general health status during the last month?” Subsequently, participants were dichotomized, according to their responses. Those who self-reported “excellent,” “very good,” or “good” were regarded as having fine SRHS, while those who reported “a little poor” or “poor” were regarded as having deteriorated SRHS.

### Restricted physical functional capacity

Restricted physical functional capacity was assessed using the Scale of Independence in Daily Living for the Disabled Elderly published by the Ministry of Health, Labour and Welfare, Japan [26]. Functional capacity impairment is a multidimensional concept that includes sensory loss, impaired mobility, vascular problems, gait impairments, difficulties with activities of daily living (ADLs), and disturbances in bodily systems [27]. A correlation between the bad scale scores and poor ADLs was confirmed [28]. In the present study, participants self-assessed their functional capacity by choosing the most appropriate one from nine options ranging from having no physical disability to being bed-bound. Participants who did not choose the option “I have no physical disability currently for daily living,” were regarded as having restricted physical functional capacity. The other eight options included “I have some disability, I do not need any care to live independently, and (1) I can go out of my house alone using public transportation or (2) I can go out by myself only within the neighborhood,” “I can live independently in my house without care, I need care to go out of my house, and (3) I spend the daytime off the bed or (4) I spend most of the daytime sleeping on and off in bed,” “I need care to live in my house, I can keep a sitting position by myself although I usually spend the daytime on the bed, and (5) I can move to a wheelchair by myself or (6) I cannot move to a wheelchair by myself,” and “I spend the whole day on the bed, I need care with excreting, eating, and dressing, and (7) I can roll over in bed or (8) I cannot roll over in bed by myself.”

### Depressive symptoms

In the present study, we used a modified 11-item Center for Epidemiological Studies Depression (CES-D) Scale [29, 30]. A score of 8 or higher was regarded as presence of depressive symptoms.

### Happiness

A single question—“How happy do you feel about your life?”—was used to assess happiness. Participants were dichotomized according to their responses; those who replied “very happy” or “happy” were regarded as experiencing happiness, while those who replied “neither happy nor unhappy” or “unhappy” were not.

### Sociodemographic and health-related backgrounds

We assessed participants’ age, occupation, type of employment (regular, irregular [part-time worker, contract employee, temporary staff], self-employed, or businesspeople), educational background (junior or senior high school, university, junior college, or vocational school), yearly household income (< 3, 3–6, 6–9, 9 ≤ million Japanese Yen [JPY]), current use of prescription medicine, social support, perceived stress, body mass index (calculated based on self-reported height and weight), smoking status (never, ex-, or current smokers), and alcohol drinking status (never, ex-, or current drinkers). As of October 2020, 100 JPY is approximately equivalent to 0.9 USD. We asked the subjects whether they were using prescription medicine for hypertension, dyslipidemia, diabetes, gout, osteoporosis, stroke, depression, blood coagulation, and other diseases. Social support was assessed with the ENRICHD Social Support Instrument (ESSI) [31, 32]. A higher total score indicates higher availability of social support. Perceived stress during the last month was assessed with the four-item Perceived Stress Scale (PSS-4) [33, 34]. A higher total score indicates higher perceived stress. The amounts of alcohol consumption per day (≤ or > 23 g a day) of current drinkers were calculated, using their self-reports on the type and amount of alcoholic drinks they were daily consuming.

### Statistical analysis

We summarized participants’ sociodemographic and health-related backgrounds and compared them by cancer history using a *t* test and chi-square test. Residual analysis was applied when chi-square test found a significant statistical difference. Multivariable logistic regression analysis was used to calculate odds ratios (ORs) and 95% confidence intervals (CIs) of working cancer survivors compared to cancer-free workers for restricted functional capacity, SRHS, the presence of depressive symptoms, and happiness. Two multivariable models were constructed. In Model 1, we adjusted for age and resident area. In Model 2, we additionally adjusted for type of employment, educational background, yearly household income, current use of prescription medicine, ESSI and PSS-4 scores, body mass index, and smoking and drinking statuses. These factors are supposed to affect functional capacity, SRHS, depressive symptoms, and happiness [5, 35–40]. We performed all the statistical analyses separately by gender since the existing sources show the cancer incidence and the frequency of cancer sites differing greatly by gender [1]. All analyses were conducted using SAS for Windows, version 9.4 (SAS/STAT 14.3; SAS Institute, Cary, NC, USA).

## Results

Of 28,311 men and 26,068 women, 977 (3.5%) and 1267 (4.9%) were working cancer survivors, respectively. There were more working cancer survivors among women than men (*p* < 0.001). The most frequent response for the cancer site was “other” (32.1% in men and 44.0% in women). Other than that, frequent cancer sites were stomach (25.7%), colorectum (22.2%), prostate (13.5%), lung (7.2%), and liver (3.4%) for men and breast (38.2%), colorectum (9.0%), stomach (7.9%), lung (3.9%), and liver (0.6%) for women. In the present study, 3.9% of male and 3.3% of female cancer survivors declared two or more cancer sites.

Participants’ sociodemographic and health-related backgrounds are summarized in Table 1 and Supplementary Table 1. For both genders, working cancer survivors were older, self-employed or businesspeople more frequently, using prescription medicine more frequently, and quit smoking and drinking more frequently than cancer-free workers. Only in men, working cancer survivors included blue-collar workers more frequently, were less educated, had lower household income, and presented higher ESSI scores and lower PSS-4 scores than cancer-free workers.

**Table 1:** Sociodemographic and health-related backgrounds of working cancer survivors and cancer-free workers according to sex, JPHC-NEXT Study, 2011–2016, Japan

Characteristic	Men (*n* = 28,311)			Women (*n* = 26,068)		
	Working cancer survivors (*n* = 977)	Cancer-free workers (*n* = 27,334)	*p* value	Working cancer survivors (*n* = 1267)	Cancer-free workers (*n* = 24,801)	*p* value
Age (mean, standard deviation)	58.4 (5.8)	53.2 (7.5)	< 0.001	54.5 (6.8)	52.4 (7.2)	< 0.001
Occupation^a)^						
White-collar	569 (58.2)	16,950 (62.0)	0.010	934 (73.7)	18,087 (72.9)	0.618
Blue-collar	403 (41.2)	10,126 (37.0)		327 (25.8)	6544 (26.4)	
Type of employment						
Regular	418 (42.8)***	15,203 (55.6)	< 0.001	452 (35.7)	8876 (35.8)	0.023
Irregular	176 (18.0)**	3920 (14.3)		534 (42.1)*	11,155 (45.0)	
Self-employed/ businesspeople	383 (39.2)***	8211 (30.0)		281 (22.2)**	4770 (19.2)	
Educational background						
Junior/senior high school	641 (65.6)	16,983 (62.1)	0.028	749 (59.1)	14,391 (58.0)	0.443
University, junior college, vocational school	336 (34.4)	10,351 (37.9)		518 (40.9)	10,410 (42.0)	
Household income (million Japanese yen)						
Less than 3	280 (28.7)**	6721 (24.6)	0.010	432 (34.1)	9149 (36.9)	0.254
3 through 6	383 (39.2)**	11,872 (43.4)		486 (38.4)	9071 (36.6)	
6 through 9	205 (21.0)	5949 (21.8)		222 (17.5)	4177 (16.8)	
9 or greater	109 (11.2)	2792 (10.2)		127 (10.0)	2404 (9.7)	
Use of prescription medicine	575 (58.9)	9969 (36.5)	< 0.001	651 (51.4)	7938 (32.0)	< 0.001
Social support (ESSI score)	27.5 (6.4)	26.2 (7.2)	0.002	27.2 (6.4)	27.1 (6.3)	0.952
Perceived stress (PSS-4 score)	6.8 (2.6)	7.0 (2.6)	< 0.001	7.5 (2.7)	7.5 (2.7)	0.693
Body mass index	23.3 (3.2)	24.2 (15.1)	0.066	22.3 (3.5)	22.9 (27.7)	0.376
Smoking						
Current smoker	284 (29.1)***	10,654 (39.0)	< 0.001	126 (9.9)	2533 (10.2)	0.035
Ex-smoker	552 (56.5)***	11,226 (41.1)		162 (12.8)**	2602 (10.5)	
Never smoker	141 (14.4)***	5454 (20.0)		979 (77.3)	19,666 (79.3)	
Alcohol drinking						
Current drinker (> 23 g/day)	457 (46.8)	12,729 (46.6)	< 0.001	102 (8.1)	2193 (8.8)	< 0.001
Current drinker (≤ 23 g/day)	319 (32.7)*	9994 (36.6)		546 (43.1)	11,015 (44.4)	
Ex-drinker	90 (9.2)***	812 (3.0)		51 (4.0)***	558 (2.2)	
Never drinker	111 (11.4)*	3799 (13.9)		568 (44.8)	11,035 (44.5)	

ESSI: ENRICHD Social Support Instrument. PSS-4: 4-item Perceived Stress Scale

Figures are presented as the number and proportion (%), except for age

Asterisks *, **, and *** indicate *p* values of less than 0.05, 0.01, and 0.001, respectively, by residual analysis

^a)^The statistic excluded those who were dedicated to two or more occupations: 5 (0.5%) male working cancer survivors, 258 (0.9%) male cancer-free workers, 6 (0.5%) female working cancer survivors, and 170 (0.7%) female cancer-free workers

Both in men (Table 2) and women (Table 3), working cancer survivors reported deteriorated SRHS and restricted physical functional capacity more frequently than cancer-free workers. There was no significant difference in the prevalence of participants with the presence of depressive symptoms between working cancer survivors and cancer-free workers both in men and women. Working cancer survivors were experiencing happiness more frequently than cancer-free workers in men, but not in women.

**Table 2:** Adjusted odds ratios (AORs) of male working cancer survivors (*n* = 977) compared to cancer-free workers (*n* = 27,334), in terms of self-rated health status (SRHS), restricted physical functional capacity, depressive symptoms, and happiness, JPHC-NEXT Study, 2011–2016, Japan

Dependent variable	*N* (%)	AOR (95% CI)	
		Model 1	Model 2
Deteriorated SRHS			
Cancer-free workers	3775 (13.8)	1	1
Working cancer survivors	208 (21.3)	1.79 (1.53–2.10)***	1.64 (1.39–1.95)***
Restricted physical functional capacity			
Cancer-free workers	715 (2.6)	1	1
Working cancer survivors	66 (6.8)	2.15 (1.65–2.80)***	1.76 (1.34–2.32)***
Depressive symptoms			
Cancer-free workers	6266 (22.9)	1	1
Working cancer survivors	211 (21.6)	1.11 (0.95–1.30)	1.17 (0.97–1.41)
Happiness			
Cancer-free workers	19,612 (71.7)	1	1
Working cancer survivors	755 (77.3)	1.26 (1.09–1.47)**	1.21 (1.01–1.45)*

*CI*, confidence interval. Adjusted odds ratios were calculated with multiple logistic regression analysis. Asterisks *, **, and *** indicate *p* values of less than 0.05, 0.01, and 0.001, respectively. In Model 1, we adjusted for age and resident area. In Model 2, in addition to the factors in Model 1, we adjusted for type of employment, educational background, yearly household income, use of prescription medicine, scores of the ENRICHD Social Support Instrument and the 4-item Perceived Stress Scale, body mass index, smoking, and alcohol drinking

**Table 3:** Adjusted odds ratios (AORs) of female working cancer survivors (*n* = 1267) compared to cancer-free workers (*n* = 24,801), in terms of self-rated health status (SRHS), restricted physical functional capacity, depressive symptoms, and happiness, JPHC-NEXT Study, 2011–2016, Japan

Dependent variable	*N* (%)	AOR (95% CI)	
		Model 1	Model 2
Deteriorated SRHS			
Cancer-free workers	4336 (17.5)	1	1
Working cancer survivors	301 (23.8)	1.52 (1.33–1.74)***	1.34 (1.16–1.54)***
Restricted physical functional capacity			
Cancer-free workers	484 (2.0)	1	1
Working cancer survivors	62 (4.9)	2.33 (1.77–3.06)***	2.06 (1.56–2.71)***
Depressive symptoms			
Cancer-free workers	7068 (28.5)	1	1
Working cancer survivors	380 (30.0)	1.12 (0.99–1.27)	1.06 (0.91–1.23)
Happiness			
Cancer-free workers	18,581 (74.9)	1	1
Working cancer survivors	964 (76.1)	1.05 (0.92–1.20)	1.12 (0.96–1.31)

*CI*, confidence interval. Adjusted odds ratios were calculated with multiple logistic regression analysis. Asterisk *** indicates *p* values of less than 0.001. In Model 1, we adjusted for age and resident area. In Model 2, in addition to the factors in Model 1, we adjusted for type of employment, educational background, yearly household income, use of prescription medicine, scores of the ENRICHD Social Support Instrument and the 4-item Perceived Stress Scale, body mass index, smoking, and alcohol drinking

## Discussion

We described the prevalence of deteriorated SRHS, restricted physical functional capacity, the presence of depressive symptoms, and happiness, and compared them between working cancer survivors and cancer-free workers. Working cancer survivors were self-employed or businesspeople more frequently than cancer-free workers. In men, blue-collar workers were more frequent in working cancer survivors than in cancer-free workers. Experiencing cancer could perhaps change the way of employment and working, although a cross-sectional examination cannot conclude the causality. Working cancer survivors exhibited a higher prevalence of deteriorated SRHS and restricted physical functional capacity than cancer-free workers. Meanwhile, there was no significant difference in the prevalence of the presence of depressive symptoms between working cancer survivors and cancer-free workers. Working cancer survivors felt happiness more frequently than cancer-free workers in men. A strength of the present study is that we indicated working cancer survivors’ SRHS, physical functional capacity, depressive symptoms, and happiness and compared to cancer-free workers at the national level by analyzing the nationwide general population sample.

### SRHS

The prevalence of deteriorated SRHS in all the subjects was approximately 15%. It is consistent with a study of another Japanese general population sample of the same age [40].

Deteriorated SRHS was reported more frequently in working cancer survivors in the present study. This is concordant with previous studies with smaller-sized samples that showed a significant association between having cancer history and poor SRHS [22, 23]. The present study had a large sample size, which made the association more prominent. The present findings confirmed that, even if cancer survivors were working, they reported deteriorated SRHS more often than cancer-free workers. Although the cross-sectional nature of the present study would not allow interpretation of causality, the present findings would suggest that working is not the absolute solution to improve the SRHS of cancer survivors. It was reported that poor SRHS increased the mortality of cancer survivors [9, 10]. Thus, continuous support would be necessary to improve cancer survivors’ SRHS and prognosis, even while they are working.

### Restricted physical functional capacity

Restricted physical functional capacity was found more often in cancer survivors than in cancer-free workers. This finding is concordant with a previous Japanese study [22]. It is suggested that some cancer survivors’ physical functional capacity remained impaired even while they were working.

The present finding would partly be supported by the existing literature. Reviews reported that breast cancer patients could have decreased strength, mobility, fatigue, and difficulties with physical tasks [18, 41, 42]. Oncotherapy could lead to a long-term decrease in the physical functional capacity in gastric and colorectal cancer patients [43, 44]. Chemotherapy, hormonal therapy, and radiation therapy could induce bone loss and muscle weakness in survivors of breast or prostate cancer, which were prevalent in working-age individuals [45, 46]. Oncotherapy could also cause cardiovascular side-effects [47, 48] and peripheral neuropathy [49]. Such physical changes would result in restricted physical functional capacity of working cancer survivors.

Since the Scale of Independence in Daily Living for the Disabled Elderly was developed to assess the physical functional capacity of elderly people, there is little evidence on whether the scale holds the validity for the middle-aged individuals. However, the scale items are easily understandable for the working-age participants to respond regarding their physical function.

### Presence of depressive symptoms

The prevalence of those having depressive symptoms was approximately 20% in men and 25% in women in the present study. The present findings, i.e., the prevalence of those having depressive symptoms and its association with cancer history, little changed even when the cut-off score of our modified CES-D scale was set at 7 or higher as a research group previously proposed (data not shown) [50]. Previous studies addressing full-time workers in Japan, who were supposed to feel very stressed, found a higher prevalence of CES-D-assessed depressive symptoms, with 25% and 34% in men and women, respectively, in one study [51], and 30% (male subjects: 73%) in another [52]. We thus believe our modified CES-D scale appropriate for evaluating the prevalence of those having depressive symptoms. However, a study limitation is that we have not yet validated our scale.

The prevalence of the presence of depressive symptoms did not differ between working cancer survivors and cancer-free workers in the present study. Previous studies that compared the prevalence between working and non-working cancer survivors exhibited inconsistent results [24]. A study of breast cancer survivors in the USA reported that the prevalence of those having depressive symptoms assessed by the CES-D did not differ between those who were and were not working [53]. In contrast, the prevalence reportedly differs between employment status in a study of hepatocellular carcinoma survivors in Japan [19].

The lack of information about the date of cancer diagnosis led to heterogeneous duration of cancer survivorship among the study participants, which might account for the insignificant difference in the prevalence of depression symptoms between working cancer survivors and cancer-free workers. Depression prevalence in cancer survivors has been shown to decrease over time after cancer diagnosis [24, 39, 54]. In addition, the prevalence of depressive symptoms in working cancer survivors might be underestimated. Some cancer patients with depressive symptoms might not have been willing to participate in the survey or might have died since depression was related to increased all-cause mortality in cancer survivors [13]. This could have decreased the prevalence of depressive symptoms in working cancer survivors, diminishing the difference from that in cancer-free workers.

### Happiness

Over 70% of the participants felt happiness in the present study. A similar proportion was reported in previous studies conducted in occupational settings in Japan [22, 55]. In general, women in Japan are more likely to report their feeling of happiness than men [56]. This was true for cancer-free workers in the present study; however, the trend was reversed for working cancer survivors.

In the present study, only male cancer survivors felt happiness more frequently than cancer-free workers. It was suggested that a role as a breadwinner was associated with happiness in men in Japan [56]. In that context, working might have contributed to increasing happiness of male working cancer survivors in Japan. In contrast, a Korean study reported that having purpose and hope in life was but the working status was not associated with happiness among breast cancer survivors [57]. The latter finding is concordant with our finding. Working might not instantly increase happiness among female cancer survivors.

It was reported in the USA and Brazil that cancer survivors were happier than the general population after adjustment for age, sex, and other demographic characteristics [58, 59]. In contrast, a previous Japanese study with a smaller-sized sample found no significant difference in the prevalence of feeling happiness between working cancer survivors and cancer-free workers in both male and female local government employees [22].

A study limitation is that we only assessed happiness with a single question—“How happy do you feel about your life?”—which may have been too simple to assess happiness. Perception of happiness consists of various factors such as personality, socioeconomic status, social network, time use and activities, stress exposure, and marital status and family [5]. Thus, previous studies used structured questionnaires such as the Oxford Happiness Questionnaire [60] and the Pemberton Happiness Index [61].

### Study limitations

One of the limitations of this study is related to the study design: of eligible cancer survivors, those who were active enough to respond to the questionnaire at the time of the survey participated in the present study. Deteriorated SRHS, restricted physical functional capacity, depressive symptoms, and unhappiness of working cancer survivors might have been underestimated if working cancer survivors had participated less frequently than cancer-free workers, for instance, due to health concerns.

Another limitation is that, as already mentioned, our questionnaire items were incomplete to specify the duration of suffering from cancer, the cancer sites, and the cancer treatment. The subjects with “other cancer” must have included those with prostate, uterus, and thyroid cancers to some extent. A discussion we cannot conclude is whether the present findings could have been varied if the proportion of cancer sites had been different. For example, working cancer survivors could have experienced deteriorated SRHS, restricted physical functional capacity, depressive symptoms, and unhappiness more frequently if the proportion of prostate, uterus, and thyroid cancers, which generally expect a relatively good prognosis, had been lower. The incomplete data regarding the duration of suffering from cancer, the cancer sites, and the cancer treatment limit the discussion of their effect on working cancer survivors’ SRHS, physical functional capacity, depressive symptoms, and happiness. However, our analysis of the nationwide data, which were heterogeneous in terms of the duration of suffering from cancer, the cancer sites, and the cancer treatment, enables us to clarify working cancer survivors’ SRHS, physical functional capacity, depressive symptoms, and happiness and to compare to cancer-free workers at the national level. It contributes to revealing the national circumstance. Our study’s purpose was not to examine the effects of a certain type of cancer on working cancer survivors’ physical and mental characteristics.

## Conclusions

Even though cancer survivors were working, compared to workers without cancer history, they had a higher prevalence of deteriorated SRHS and restricted physical functional capacity. Continuous support to improve the SRHS and physical functional capacity of cancer survivors would be necessary for their better health even while they are working.

## Supplementary Information

ESM 1 (PDF 142 kb)

## Data Availability

Investigators are granted access to JPHC-NEXT data after approval of the JPHC-NEXT Steering Committee and the Institutional Review Board. Please visit the following website for more information: https://epi.ncc.go.jp/jphcnext/en/access/index.html
