# Supplementary material for: Working cancer survivors’ physical and mental characteristics compared to cancer-free workers in Japan: a nationwide general population-based study
Source: J Cancer Surviv. 2021 Jan 12;15(6):912–21. doi: 10.1007/s11764-020-00984-7 (PMC8519890; doi:10.1007/s11764-020-00984-7)
Supplement: Supplementary file 1 — (PDF 142 kb) [file 11764_2020_984_MOESM1_ESM.pdf]

Supplementary Table 1. Occupation of working cancer survivors and cancer-free workers according to sex, JPHC-NEXT Study, 2011 – 2016, Japan

Characteristic	Men (n = 28,311)			Women (n = 26,068)		
	Working cancer survivors (n = 977)	Cancer-free workers (n = 27,334)	p-value	Working cancer survivors (n = 1,267)	Cancer-free workers (n = 24,801)	p-value
Occupation						
Professional/technical work	203 (20.8)*	6,619 (24.2)	<0.001	316 (24.9)	6,117 (24.7)	0.481
Administrative work	145 (14.8)	3,762 (13.8)		43 (3.4)	699 (2.8)	
Clerk	69 (7.1)	2,057 (7.5)		206 (16.3)	4,590 (18.5)	
Sales	53 (5.4)	1,537 (5.6)		106 (8.4)	1,794 (7.2)	
Service	99 (10.1)	2,975 (10.9)		263 (20.8)	4,887 (19.7)	
Security	17 (1.7)	354 (1.3)		1 (0.1)	11 (0.0)	
Agriculture/fishery	189 (19.3)***	3,685 (13.5)		130 (10.3)	2,490 (10.0)	
Transportation or telecommunications	56 (5.7)	1,594 (5.8)		6 (0.5)	153 (0.6)	
Industrial operation/ management	109 (11.2)	3,611 (13.2)		143 (11.3)	2,956 (11.9)	
Others	32 (3.3)	882 (3.2)		47 (3.7)	934 (3.8)	
Dedicated to two or more Occupations	5 (0.5)	258 (0.9)		6 (0.5)	170 (0.7)	

Figures are presented as the number and proportion (%).

* and *** indicate p-values of less than 0.05 and 0.001, respectively, by residual analysis.
